# Severe periocular ecchymosis following acupuncture therapy for dry eye: a report of two cases

**DOI:** 10.1093/jscr/rjae783

**Published:** 2025-01-31

**Authors:** Jarryl H J Tsai, Jonathan T W Au Eong, Kah-Guan Au Eong

**Affiliations:** Lee Kong Chian School of Medicine, Nanyang Technological University, 11 Mandalay Road, Singapore 308232, Singapore; Lee Kong Chian School of Medicine, Nanyang Technological University, 11 Mandalay Road, Singapore 308232, Singapore; Lee Kong Chian School of Medicine, Nanyang Technological University, 11 Mandalay Road, Singapore 308232, Singapore; International Eye Cataract Retina Center, Farrer Park Medical Center, 1 Farrer Park Station Road #14-07/08, Connexion, Singapore 217562, Singapore; Department of Ophthalmology and Visual Sciences, Khoo Teck Puat Hospital, 90 Yishun Central, Singapore 768828, Singapore

**Keywords:** periocular ecchymosis, acupuncture therapy, dry eye

## Abstract

Acupuncture is a complementary therapy used in many parts of the world to treat a variety of disorders. Two women aged 61 and 86 years old presented with painless bruising around their right eye for 3 and 10 days, respectively, following acupuncture therapy for dry eye. The younger woman was on oral acetylsalicylic acid 100 mg daily and it was her second episode of ocular bruising from a total of 20 therapy sessions. Clinical examination disclosed severe periocular ecchymosis in their right eye. There was no proptosis or globe perforation. The ecchymosis gradually resolved over a few weeks without any visual sequelae in both cases. Periocular acupuncture can cause severe periocular ecchymoses. Patients should be informed of this and other potentially sight-threatening complications associated with this form of treatment, and both acupuncture practitioners and ophthalmologists should be alert to recognize such complications early so that they can be appropriately managed.

## Introduction

The ancient practice of acupuncture originated in China and dates back 3000 years [1]. It is a well-received complementary therapy in many parts of the world, in which practitioners insert fine needles into specific points to a distance known as the therapeutic depth, treating a variety of health problems. The therapeutic depth is defined as the depth at which the needle reaches the muscular layer of the specific acupuncture point [2]. Due to the widespread use of acupuncture, its safety is an important issue that deserves close attention [3].

While acupuncture is most commonly used in the treatment of chronic, non-cancer pain in adults [4], it is also used to treat the symptoms of dry eye [5, 6]. There is scarce literature on the potential adverse effects of acupuncture therapy for dry eye. We report two cases of severe periocular ecchymosis following acupuncture therapy to highlight this potential complication.

## Case 1

A 61-year-old woman presented with painless bruising around her right eye for three days after receiving acupuncture therapy for dry eye.

Immediately after the acupuncture therapy, she noticed swelling around her right eye, which gradually progressed to bruising. She did not apply any compression to her eye. She reported no visual disturbance or pain, and still had persistent dry eye symptoms.

The patient has had acupuncture therapy weekly for a total of 20 times and this was her second episode of ocular bruising. She had experienced minor periocular bruising in her left eye during one of her earlier treatments but did not seek any medical attention from a doctor. The bruising gradually resolved spontaneously.

The patient has a history of cataract surgery in both eyes and pulmonary tuberculosis, and was on follow up for mitral valve prolapse, hypertension and hypercholesterolemia. She was on oral statin and acetylsalicylic acid 100 mg daily.

**Figure 1:**
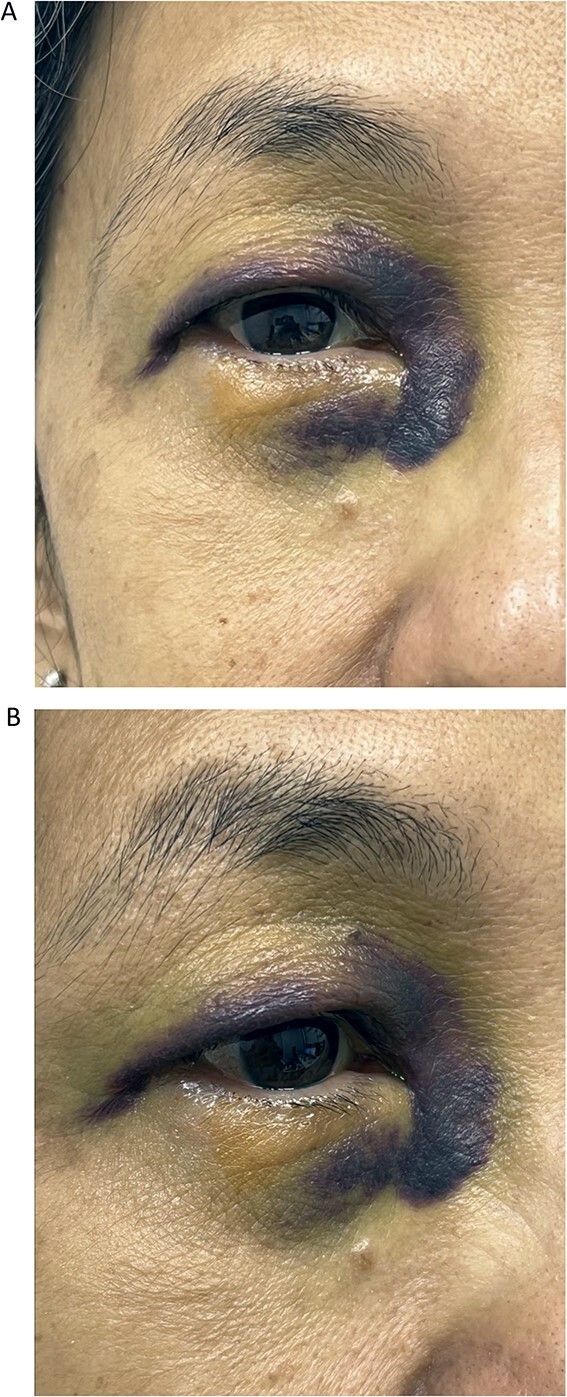
Front (A) and oblique (B) views of right eye of Case 1 showing severe periorbital ecchymosis 3 days after receiving acupuncture therapy for dry eye.

Her best-corrected visual acuity was 20/20 in both eyes. Clinical examination disclosed severe periocular ecchymosis in her right eye (Fig. 1). The extraocular movements were normal in both eyes. There was no proptosis or globe perforation. She had blepharitis and dry eye in both eyes. She was pseudophakic in both eyes and there was no other ocular pathology.

No treatment was given for the ecchymosis and it gradually resolved over a few weeks without any visual sequelae.

## Case 2

A 86-year-old woman presented with painless bruising around her right eye following acupuncture therapy for dry eye 10 days prior.

The patient had cataract surgery in both eyes 6 years earlier and had Nd:YAG laser posterior capsulotomy in her left eye one year earlier. She also had dry age-related macular degeneration in both eyes and was on oral high-dose antioxidant vitamins. She had a valvular heart disease for more than 10 years and used continuous positive airway pressure machine for obstructive sleep apnoea. She previously underwent colorectal surgery for colorectal carcinoma. She was not on any antiplatelet or anticoagulation therapy.

**Figure 2:**
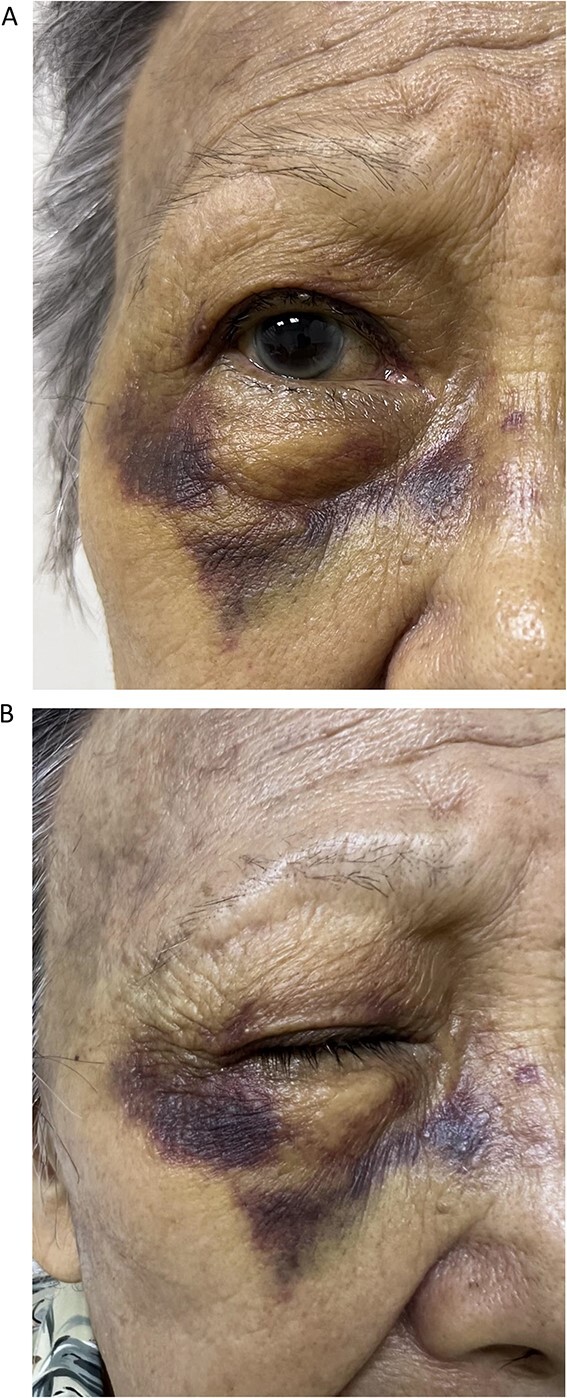
Front (A) and oblique (B) views of right eye of Case 2 showing severe periorbital ecchymosis 10 days after receiving acupuncture therapy for dry eye.

Her best-corrected visual acuity was 20/20 in both eyes. Clinical examination disclosed severe periocular ecchymosis in her right eye (Fig. 2). Her extraocular movements were normal in both eyes. There was no proptosis or globe perforation. She was pseudophakic and had dry eye, blepharitis, and drusen in both eyes.

No treatment was given for the ecchymosis and it gradually resolved over a few weeks without any visual sequelae.

## Discussion

Periocular ecchymosis is a common clinical finding most often occurring due to trauma. The highly vascularized network under the periocular skin makes it prone to bruising [7].

The periocular ecchymosis secondary to acupuncture therapy in our patients was sufficiently alarming to cause them to seek an ophthalmologist’s consultation. One of them was on antiplatelet therapy which is a risk factor for bleeding and in fact, she experienced two episodes of periocular ecchymoses from 20 acupuncture sessions. In both cases, the condition resolved gradually and was visually inconsequential. To the best of our knowledge, periocular ecchymosis due to acupuncture has not been previously reported in the literature.

Conceivably, the nature of periocular acupuncture suggests that potentially more serious ophthalmic injuries can occur, such as traumatic damage to the globe and periocular structures such as large blood vessels and extraocular muscles. In fact, a total of 14 case reports involving 15 eyes injured by periocular acupuncture were retrieved by Lee and associates in a systematic search of four databases (PubMed, MEDLINE, Embase, and KoreaMed) of English-language articles published between 1980 and 2020 (Table 1) [8]. Twelve of these eyes had penetrating globe injuries [8]. Treatments rendered included pars plana vitrectomy in six eyes (one of which underwent the procedure twice), and phacoemulsification and intraocular lens implantation in four eyes. The final visual acuity was reported in 11 of the 15 eyes and was 20/20 in one eye, 20/25 to 20/40 in six eyes, 20/60 to 20/200 in one eye, and worse than 20/200 in three eyes (including one which was reported as ‘blind’). Two eyes were reported to have recovered but the visual acuity was not reported while the visual acuity of another two eyes were unknown due to follow-up loss.

**Table 1:** Summary of patients with ophthalmic complications associated with periocular acupuncture in Lee and associates’ review (Adapted from Lee SM, Wu J, Hwang DDJ. Severe Adverse Events of Periocular Acupuncture: A Review of Cases. *Korean J Ophthalmol*. 2023;**37**:255–65. https://doi.org/10.3341/kjo.2022.0111)

Patient number	Number of eyes affected	Country of report	Age (years)	Sex	Reason for acupuncture	Complication(s)	Treatment	Outcome (visual acuity)
1	1	China	20	Male	Traumatic mydriasis	Blepharospasm, lagophthalmos	Warm compress	Recovered (visual acuity not reported)
2	1	China	28	Female	Chronic conjunctivitis	Eye hematoma, medial palpebral artery trauma	Cold and warm compress	Recovered (visual acuity not reported)
3	1	China	52	Male	Blepharospasm	Retinal detachment	Pars plana vitrectomy	Recovered (20/100)
4	1	China	63	Female	Ptosis	Traumatic cataract	Refused operation	Unknown due to follow-up loss
5	1	China	62	Male	Lateral rectus palsy	Optic atrophy due to pressure from retrobulbar hemorrhage	Lateral canthotomy	Blindness
6	1	Canada	67	Male	Glaucoma	Retinal tear and vitreous hemorrhage	Pars plana vitrectomy	Recovered (20/40)
7	1	Korea	42	Female	Blepharospasm	Retinal tear and vitreous hemorrhage	Pars plana vitrectomy	Recovered (20/20), visual field defect
8	1	USA	42	Female	Hemifacial spasm	Retinal tear and subretinal track	Retinal laser	Unknown due to follow-up loss
9	1	China	58	Male	Cerebral infarction	Traumatic cataract, corneal and iris perforation	Phacoemulsification, anterior vitrectomy, intraocular lens implantation	Recovered (20/30)
10	1	Korea	62	Male	Chronic conjunctivitis, glaucoma	Retinal tear and hemorrhage, vitreous hemorrhage, suspected endophthalmitis	Pars plana vitrectomy, intravitreal antibiotics injection	Recovered (20/25), visual field defect
11	1	Taiwan	72	Female	Glaucoma	Retinal hole, retinal hemorrhage	Retinal endolaser	Recovered (20/25)
12	1	Malaysia	63	Female	Glaucoma	Subconjunctival hemorrhage	Observation	Recovered (20/600)
13	1	Canada	49	Female	Headache	Subconjunctival hemorrhage, 4+ cell and fibrin in anterior chamber, vitreous hemorrhage, subretinal hemorrhage	Pars plana vitrectomy twice, phacoemulsification and intraocular lens implantation, intravitreal methotrexate injection	Recovered (20/40)
14	2	Korea	58	Female	Glaucoma prevention	Both eyes had subconjunctival hemorrhage, cells in anterior chamber, vitreous hemorrhage	Pars plana vitrectomy in right eye, phacoemulsification and intraocular lens implantation in both the eyes	Recovered (right eye, 20/40; left eye, 20/2000

A 2010 systematic review of Chinese-language literature from three Chinese databases (Chinese Biomedical Literature Database [1980-2009], Chinese Journal Full-text Database [1980-2009] and Weipu Journal Database [1989-2009]) on acupuncture-related adverse events identified five articles that reported ophthalmic injuries [3]. There were three cases of orbital hemorrhages, one traumatic cataract, one oculomotor nerve injury, one retinal puncture and one case of optic atrophy accompanied by hemorrhage and traumatic cataract [3].

Interestingly, at least three of the 15 eyes in Lee and associates’ review were injured by unlicensed practitioners [8]. This highlights the importance of proper training, accreditation, regulation and enforcement in improving the quality and safety of acupuncture treatment and in protecting the public from unlicensed practitioners.

Despite the risks, there is some evidence that supports the use of acupuncture for dry eye treatment [5, 9]. A recent meta-analysis concluded that acupuncture can effectively improve the results of tear breakup time, Schirmer test, chronic fatigue syndrome and score of symptoms for dry eye patients [10]. However, its therapeutic mechanisms remain to be clarified. Some postulated mechanisms include promoting secretion of tears, reducing pain and inflammation, increasing ocular blood flow, and regulating the immune and nervous system [10].

As acupuncture around the periorbital area poses a risk of ophthalmic injury, it is imperative for both acupuncture practitioners and ophthalmologists to be aware of these potential complications and to recognize them early so that such cases can be managed expeditiously and appropriately. Patients undergoing periocular acupuncture should also be made aware of its potential complications during consent taking before the treatment is given.

## Conclusion

Periocular acupuncture can cause severe periocular ecchymosis. Patients should be informed of this and other potentially sight-threatening complications associated with this form of treatment, and both acupuncture practitioners and ophthalmologists should be alert to recognize such complications early so that they can be appropriately managed.
